# Antinociceptive Efficacy of the µ-Opioid/Nociceptin Peptide-Based Hybrid KGNOP1 in Inflammatory Pain without Rewarding Effects in Mice: An Experimental Assessment and Molecular Docking

**DOI:** 10.3390/molecules26113267

**Published:** 2021-05-28

**Authors:** Maria Dumitrascuta, Marcel Bermudez, Olga Trovato, Jolien De Neve, Steven Ballet, Gerhard Wolber, Mariana Spetea

**Affiliations:** 1 Department of Pharmaceutical Chemistry, Institute of Pharmacy, Center for Molecular Biosciences Innsbruck (CMBI), University of Innsbruck, Innrain 80-82, 6020 Innsbruck, Austria; maria.dumitrascuta@student.uibk.ac.at (M.D.); olga.trovato@uibk.ac.at (O.T.); 2 Institute of Pharmacy, Freie Universität Berlin, Königin-Luise-Str. 2+4, D-14195 Berlin, Germany; m.bermudez@fu-berlin.de (M.B.); gerhard.wolber@fu-berlin.de (G.W.); 3 Research Group of Organic Chemistry, Departments of Chemistry and Bioengineering Sciences, Vrije Universiteit Brussel, Pleinlaan 2, B-1050 Brussels, Belgium; jolien.de.neve@vub.be (J.D.N.); steven.ballet@vub.be (S.B.)

**Keywords:** µ-opioid receptor, nociceptin receptor, multitarget ligands, inflammatory pain, side effects, reward, molecular docking

## Abstract

Opioids are the most effective analgesics, with most clinically available opioids being agonists to the µ-opioid receptor (MOR). The MOR is also responsible for their unwanted effects, including reward and opioid misuse leading to the current public health crisis. The imperative need for safer, non-addictive pain therapies drives the search for novel leads and new treatment strategies. In this study, the recently discovered MOR/nociceptin (NOP) receptor peptide hybrid KGNOP1 (H-Dmt-*D*-Arg-Aba-β-Ala-Arg-Tyr-Tyr-Arg-Ile-Lys-NH_2_) was evaluated following subcutaneous administration in mouse models of acute (formalin test) and chronic inflammatory pain (Complete Freund’s adjuvant-induced paw hyperalgesia), liabilities of spontaneous locomotion, conditioned place preference, and the withdrawal syndrome. KGNOP1 demonstrated dose-dependent antinociceptive effects in the formalin test, and efficacy in attenuating thermal hyperalgesia with prolonged duration of action. Antinociceptive effects of KGNOP1 were reversed by naltrexone and SB-612111, indicating the involvement of both MOR and NOP receptor agonism. In comparison with morphine, KGNOP1 was more potent and effective in mouse models of inflammatory pain. Unlike morphine, KGNOP1 displayed reduced detrimental liabilities, as no locomotor impairment nor rewarding and withdrawal effects were observed. Docking of KGNOP1 to the MOR and NOP receptors and subsequent 3D interaction pattern analyses provided valuable insights into its binding mode. The mixed MOR/NOP receptor peptide KGNOP1 holds promise in the effort to develop new analgesics for the treatment of various pain states with fewer MOR-mediated side effects, particularly abuse and dependence liabilities.

## 1. Introduction

Effective pain treatment, particularly chronic pain, remains an unmet medical need at the beginning of the 21st century. While opioid-based pharmacotherapy is still the most powerful strategy for the treatment of moderate to severe pain, the risk–benefit ratio is suboptimal because of frequent and serious side effects [1]. The dramatic increase in the medical use and misuse of opioids with the concurrent rising number of overdose deaths and opioid-use disorders has led to the current opioid crisis [2,3]. Hence, research efforts are needed to overcome the limitations of present therapies with the aim to improve treatment efficacy and reduce complications.

The µ-opioid receptor (MOR) is a member of the opioid system, together with the δ- (DOR), κ- (KOR), and nociceptin (NOP) receptors and their endogenous peptides [4], modulating both nociception and reward systems [5]. The majority of clinically used opioids are agonists to the MOR, and these are vastly misused and abused [1]. Alternative chemical and pharmacological strategies are therefore evaluated to mitigate the deleterious effects of opioids, amongst which are multifunctional ligands, G protein-biased agonists, peripherally restricted opioids, and abuse-deterrent formulations of existing opioids [6,7,8,9,10]. Furthermore, the available structures of the MOR provide a valuable opportunity for computational drug design and discovery [11,12,13].

The concept of ‘one molecule, multiple targets’ received increased attention in the opioid research over the past years as a promising approach for the discovery of effective and safer opioids [6,8,14,15,16,17,18]. Bifunctional ligands targeting the MOR simultaneously with other (opioid/non-opioid) neurotransmitter systems implicated in pain processing and/or opioid-induced side effects are of particular interest [8,19,20,21].

The recently discovered bifunctional peptide-based hybrid KGNOP1, H-Dmt-*D*-Arg-Aba-β-Ala-Arg-Tyr-Tyr-Arg-Ile-Lys-NH_2_ (Dmt: 2′,6′-dimethyl-L-Tyr; Aba: 4-amino-tetrahydro-2-benzazepinone), combines a MOR pharmacophore, H-Dmt-*D*-Arg-Aba-β-Ala-NH_2_, and a NOP receptor pharmacophore, H-Arg-Tyr-Tyr-Arg-Ile-Lys-NH_2_ [22]. KGNOP1 was reported to produce effective antinociception in rodent models of acute nociception and neuropathic pain with a lower propensity for respiratory depression than conventional opioids [22,23,24]. In this study, we further investigated the in vivo effects of KGNOP1 in mouse models of acute and chronic inflammatory pain after subcutaneous (s.c.) administration and assessed potential opioid liabilities for locomotor dysfunction and rewarding and withdrawal effects after chronic treatment, in comparison to the clinically relevant morphine. Furthermore, we provide new understanding on KGNOP1’s mechanism of action and report the first structure-based investigation on KGNOP1 binding to the structures of the MOR [12] and NOP receptors [25].

## 2. Results

### 2.1. Pharmacological Properties of KGNOP1 In Vitro

**Figure 1:**
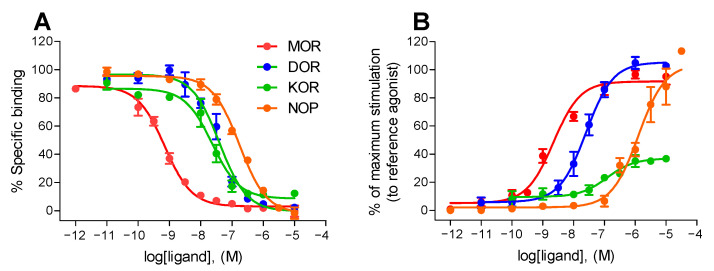
In vitro activity profile of KGNOP1 to the human opioid receptors. (**A**) Binding curves of KGNOP1 to the human opioid receptors determined in competitive radioligand binding assays. Concentration-dependent inhibition by KGNOP1 of [^3^H]DAMGO (MOR), [^3^H]diprenorphine (DOR), [^3^H]U69,593 (KOR), and [^3^H]Nociceptin (NOP receptor) binding to membranes of CHO cells stably expressing the human opioid receptors (CHO-hOR). (**B**) Concentration-dependent stimulation of [^35^S]GTPγS binding by KGNOP1 in the [^35^S]GTPγS binding assays using CHO cell membranes stably expressing the human opioid receptors. Data are presented as percentage stimulation relative to the maximum effect of reference full agonists DAMGO (MOR), DPDPE (DOR), U69,593 (KOR), and nociceptin (NOP receptor) (as 100%). Values are expressed as the mean ± SEM (n = 3–4 independent experiments performed in duplicate).

We re-evaluated the binding properties of KGNOP1 to the human MOR, DOR, KOR, and NOP receptors by radioligand binding to membrane preparations from Chinese hamster ovary (CHO) cells overexpressing recombinant receptors, as described previously [26,27] (Figure 1A). KGNOP1 demonstrated subnanomolar affinity (K_i_ = 0.42 nM) for the MOR, while having lower binding affinities for the DOR and KOR (Table 1). KGNOP1 also bound to the NOP receptor (K_i_ = 141 nM), although with reduced affinity in comparison with the other opioid receptors, as determined in competitive radioligand binding assays, and also aligned with previously reported data [22].

Initial data on the in vitro functional activity of KGNOP1 were reported in guinea pig ileum and mouse vas deferens bioassays [22], with KGNOP1 depicted as a potent MOR agonist. In the current study, we assessed the in vitro activity of KGNOP1 to the human opioid receptors using the guanosine 5′-O-(3-[^35^S]thio)triphosphate ([^35^S]GTPγS) functional assay with membranes of CHO cells expressing the MOR, DOR, KOR, or NOP receptors [26], with agonist potency (ED_50_) and efficacy (E_max_) values shown in Table 1. Stimulation of the [^35^S]GTPγS binding induced by KGNOP1 was compared to the effect of reference full agonists [*D*-Ala^2^,*N*-Me-Phe^4^,Gly-ol^5^]enkephalin (DAMGO, MOR), [*D*-Pen^2^,*D*-Pen^5^]enkephalin (DPDPE, DOR), U69,593 (KOR), and nociceptin (NOP receptor). As shown in Figure 1B, KGNOP1 produced a concentration-dependent increase in the [^35^S]GTPγS binding with the highest potency and full efficacy to the MOR. At the DOR and KOR, KGNOP1 showed reduced potencies, while displaying full and partial agonism, respectively (Table 1). Furthermore, KGNOP1 showed full efficacy for the G protein activation using the [^35^S]GTPγS binding assay in NOP receptor-expressing CHO cells (E_max_ = 99%), albeit with a very low potency (Figure 1B, Table 1).

### 2.2. KGNOP1 Significantly Attenuates Pain Behavior in the Mouse Formalin Test

**Figure 2:**
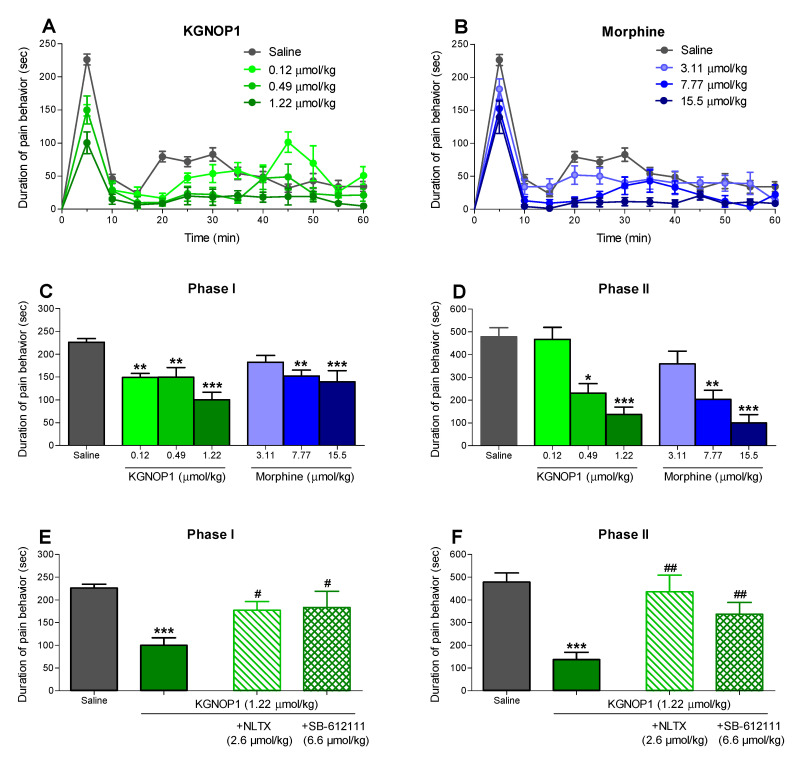
Antinociceptive effect of KGNOP1 and morphine after s.c. administration in the formalin test in mice. Groups of mice received s.c. saline (control) or different doses of KGNOP1 (**A**,**C**) or morphine (**B**,**D**) before an intraplantar injection of the formalin solution in the right hindpaw, and the duration of pain behavior was monitored for 60 min, determined as the amount of time (in seconds, sec) each animal spent licking, biting, lifting, and flinching the formalin-injected paw. Time-course of pain behavior of mice treated with KGNOP1 (**A**) and morphine (**B**). Dose-dependent antinociceptive effect of KGNOP1 and morphine during Phase I (**C**) and Phase II (**D**) of the formalin test. Effect of pre-treatment with naltrexone (2.6 µmol/kg s.c., -15 min) or SB-612111 (6.6 µmol/kg s.c., -30 min) on the antinociceptive effect of KGNOP1 (1.22 mg/kg) during Phase I (**E**) and Phase II (**F**) of the formalin test. Data are presented as the mean ± SEM (n = 6–8 mice per group). * *p* < 0.05, ** *p* < 0.01, and *** *p* < 0.001 vs. saline group, one-way ANOVA followed by Dunnett’s post hoc test (**C**,**D**); *** *p* < 0.001 vs. saline group and ^#^ *p* < 0.05 and ^##^ *p* < 0.01 vs. KGNOP1-treated group, unpaired *t*-test.

Intraplantar administration of the formalin solution to the mouse hindpaw induces a pain response in a biphasic manner [28]. The first phase is characterized by the acute activation of nociceptors, while the second phase involves an inflammatory reaction in the peripheral injured tissue. We used this preclinical pain model for acute inflammatory pain to assess the antinociceptive effects of KGNOP1 after s.c. administration to mice, and to compare its effect to that of morphine. Systemic administration of KGNOP1 and morphine produced a dose-dependent reduction in pain behavior of formalin-injected mice, determined as the amount of time (in seconds, sec) each animal spent licking, biting, lifting, and flinching the formalin-injected paw (Figure 2A,B and Table S1), with an almost complete inhibition of the pain response counted between 15 and 60 min in mice treated with KGNOP1 (1.22 µmol/kg) and morphine (15.5 µmol/kg). Both KGNOP1 and morphine attenuated the nociceptive response during Phase I of the formalin assay with a significant effect at all tested doses of KGNOP1, and at 7.77 and 15.5 µmol/kg of morphine (Figure 2C). During Phase II, KGNOP1 and morphine also produced a dose-dependent decrease in pain response with a significant effect at 0.49 and 1.22 µmol/kg of KGNOP1, and at 7.77 and 15.5 µmol/kg of morphine (Figure 2D). The calculated antinociceptive ED_50_ values for KGNOP1 in the acute nociceptive Phase I and inflammatory Phase II of the formalin test were 1.17 µmol/kg (95% confidence limits, CL, 0.41–3.35) and 0.55 µmol/kg (95% CL, 0.22–1.33), respectively, and for morphine in Phase II was 6.44 µmol/kg (95% CL 3.20–12.7) (Table S1).

To evaluate the involvement of the MOR and/or NOP receptors in KGNOP1-induced antinociception in the formalin test, the effect of the MOR antagonist naltrexone and NOP receptor antagonist SB-612111 was tested [29,30] (Figure 2E,F). Pre-treatment of mice with naltrexone (2.6 µmol/kg, s.c.) resulted in a significant antagonism of the antinociceptive effect of KGNOP1 in both Phase I and II. The NOP receptor selective antagonist SB-612111 (6.6 µmol/kg, s.c.) also significantly reversed the antinociceptive response during the nociceptive and inflammatory phase of the formalin test (Figure 2E,F).

### 2.3. KGNOP1 Efficiently Reverses Hyperalgesia in Mice with Complete Freund’s Adjuvant-Induced Chronic Inflammatory Pain

**Figure 3:**
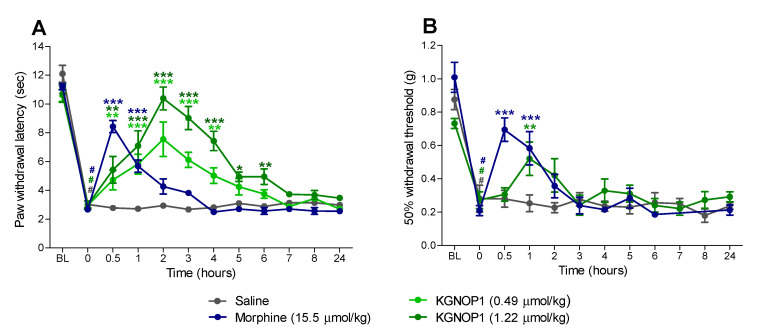
Antinociceptive effect of KGNOP1 and morphine after s.c. administration to mice with CFA-induced inflammatory pain. Groups of mice received s.c. saline (control), KGNOP1, or morphine, 72 h after i.pl injection of CFA in the right hindpaw. Paw withdrawal latencies to thermal (Hargreaves test, **A**) and mechanical stimulation (von Frey test, **B**) were determined before (baseline, BL), 72 h after CFA injection (0 h), and at different time points up to 24 h after drug administration. Data are presented as the mean ± SEM (n = 6–8 mice per group). * *p* < 0.05, ** *p* < 0.01, and *** *p* < 0.001 vs. saline group, two-way ANOVA followed by Bonferroni’s post hoc test; ^#^ *p* < 0.001 vs. baseline (BL) pre-inoculation values, paired *t*-test.

Chronic inflammatory pain was induced by injection of Complete Freund’s Adjuvant (CFA) [31] to the dorsal side of the right hindpaw, evidenced by a significant reduction at 72 h post-inoculation in paw withdrawal thresholds to thermal and mechanical stimulation (Figure 3). In this study, mice were treated s.c. with saline, KGNOP1 (0.49 and 1.22 µmol/kg), or morphine (15.5 µmol/kg), and tested for thermal and mechanical sensitivity using the Hargreaves (Figure 3A) and von Frey tests (Figure 3B), respectively.

As shown in Figure 3A and Table S2, KGNOP1 produced a dose- and time-dependent increase in the inflamed paw withdrawal latencies to thermal stimulation. Compared to saline-treated animals, both tested doses of KGNOP1 (0.49 and 1.22 µmol/kg) significantly inhibited thermal hyperalgesia from 30 min to 4 h, and 30 min to 6 h, respectively. Notable was the long duration of action of KGNOP1 in the Hargreaves test, with a dose of 1.22 µmol/kg reaching maximum efficacy at two hours, and thermal nociceptive thresholds returning to the basal values. Administration of morphine (15.5 µmol/kg) caused a significant reduction in CFA-induced thermal hyperalgesia at 30 min and one hour (Figure 3A). A fast onset of the antihyperalgesic effect of morphine was observed with a peak effect at 30 min followed by a rapid decline. Paw withdrawal thresholds to mechanical stimulation were also assessed in CFA-injected mice after s.c. administration of KGNOP1 (Figure 3B and Table S2). In the inflamed hindpaw, KGNOP1 (1.22 µmol/kg) reduced the pain response to mechanical stimulation, with a significant effect at one hour, whereas morphine (15.5 µmol/kg) showed a significant increase at 30 min and one hour, when compared to saline-treated mice (Figure 3B).

### 2.4. Chronic Administration of KGNOP1 Does Not Affect Spontaneous Locomotor Activity of Mice

**Figure 4:**
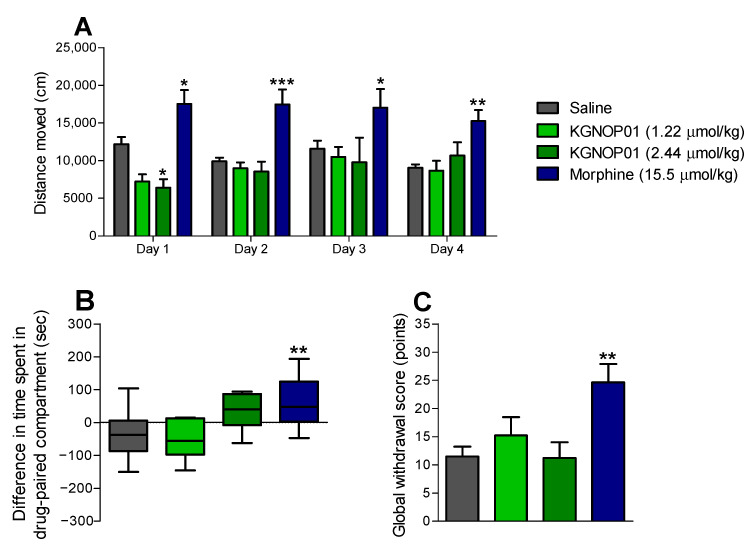
Behavioral responses induced by KGNOP1 and morphine after chronic s.c. administration to mice. (**A**) Spontaneous locomotor activity in mice treated with saline (control), KGNOP1, or morphine as distance traveled during 30 min after drug daily administration for 4 days. (**B**) Rewarding effects in mice treated with saline (control), KGNOP1, or morphine in the CPP test after drug conditioning for 4 days. (**C**) Physical dependence as naloxone-induced withdrawal syndrome in mice treated daily for 5 days with saline (control), KGNOP1, or morphine. Withdrawal was precipitated two hours after last drug administration using naloxone (1 mg/kg, s.c.) and signs of withdrawal were measured over 15 min immediately after naloxone, and a global withdrawal score was calculated. See also Figure S1. Data are presented as the mean ± SEM (n = 8 mice per group). ** *p* < 0.05, ** *p* < 0.01, and *** *p* < 0.001 vs. saline group, two-way ANOVA followed by Bonferroni’s post hoc test (**A**); * *p* < 0.05 and ** *p* < 0.01 vs. saline group, one-way ANOVA followed by Dunnett’s post hoc test (**B**,**C**).

Behavioral effects on spontaneous locomotor activity of KGNOP1 after chronic s.c. treatment to mice were assessed using the open-field test (Figure 4A). Mice were administered KGNOP1 (1.22 µmol/kg) or morphine (15.5 µmol/kg) daily for 4 days, at doses shown to be effective in producing an antinociceptive effect in mouse models of inflammatory pain, the formalin test and CFA-induced hyperalgesia (Figure 2 and Figure 3). Whereas morphine-treated animals showed a significant increase in the distance traveled compared to the saline group, already from the first day until day 4, no significant alteration in locomotion was observed in mice who were administered 1.22 µmol/kg of KGNOP1. Only at day 1, a significant decrease in locomotion was produced by KGNOP1 at a higher dose of 2.44 µmol/kg (Figure 4A).

### 2.5. KGNOP1 Does Not Induce Rewarding Effects in Mice

To address the potential rewarding effects of KGNOP1, a conditioning paradigm was performed in mice after s.c. drug administration (Figure 4B). Mice were subjected to daily treatments with KGNOP1 (1.22 µmol/kg), morphine (15.5 µmol/kg), or saline. Drug doses were selected based on the established antinociceptive efficacy in mouse models of inflammatory pain (Figure 2 and Figure 3). Additionally, the conditioned place preference (CPP) test was performed in mice receiving a higher dose of 2.44 µmol/kg of KGNOP1. Following a 4-day place conditioning paradigm, as expected, mice conditioned with morphine (15.5 µmol/kg) demonstrated a significant CPP response compared to saline-treated animals. In contrast, KGNOP1-treated mice showed no significant preference at any tested dose (Figure 4B).

### 2.6. Chronic Administration of KGNOP1 Does Not Induce Withdrawal Syndrome in Mice

To further assess the behavioral effects of KGNOP1 after chronic s.c. treatment to mice, the potential for physical dependence was determined using naloxone-precipitated withdrawal. Mice were treated daily over a 5-day period using the same drug doses as in the locomotor and CPP experiments, KGNOP1 (1.22 and 2.44 µmol/kg), morphine (15.5 µmol/kg), or saline (control). Administration of naloxone (1 mg/kg, s.c.), two hours after the last injection of drugs, induced higher scores for withdrawal signs in morphine-dependent mice, as compared to KGNOP1 and saline-treated mice (Figure S1). Analysis of the global withdrawal score revealed a significantly increased withdrawal in morphine-treated mice, but not in KGNOP1-treated, neither at 1.22 µmol/kg nor 2.44 µmol/kg, when compared to saline controls (Figure 4C).

### 2.7. Molecular Docking of KGNOP1 to the MOR and NOP Receptors

**Figure 5:**
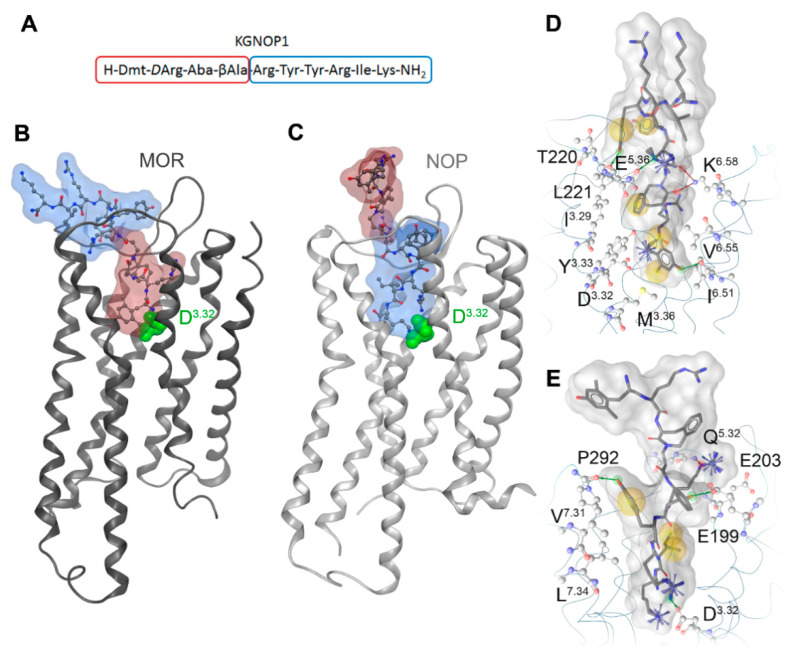
Proposed binding modes of KGNOP1 (**A**) to the active state of the MOR ((**B**); PDB entry 6DDF, chain R) and the inactive conformation of the NOP receptor ((**C**); PDB entry 5DHG, chain A). The bifunctional peptide KGNOP1 consists of a MOR-binding scaffold and a substructure binding to the NOP receptor (red and blue surface, respectively; **A**–**C**). These receptor-specific peptide parts bind in opposing orientations to the inner core region of both receptors driven by a charge interaction with D^3.32^ (green). A more detailed interaction pattern is visualized in (**D**) for the MOR-KGNOP1 complex and (**E**) for the NOP receptor-KGNOP1 complex, by illustrating hydrophobic contacts as yellow spheres, positively ionizable centers as blue stars and hydrogen bond acceptors and donors as red and green arrows, respectively.

KGNOP1 has been designed as a bifunctional peptide ligand consisting of substructures known to bind to the MOR and NOP receptors [22] (Figure 5A). With the aim of gaining molecular insights into the binding modes of KGNOP1 to the MOR and NOP receptors, we performed molecular docking studies using the active cryo-EM structure of the mouse MOR (Protein Data Bank, PDB entry: 6DDF) [12] and the reported inactive crystal structure of human NOP receptor (PDB entry: 5DHG) [25]. Each individual pharmacophore, H-Dmt-*D*-Arg-Aba-β-Ala-NH_2_ for the MOR and H-Arg-Tyr-Tyr-Arg-Ile-Lys-NH_2_ for the NOP receptor, binds to the inner core region of the respective receptor, and thereby determines the binding orientation (Figure 5B,C). The aspartic acid D^3.32^ forms charge interactions in both cases with the N-terminal α-amine of the Dmt scaffold at the MOR and with the basic amino acids Arg^8^ and Lys^10^ of KGNOP1 in the NOP receptor (Figure 5D,E). Similar to the previously reported MOR agonist KGOP01 [32], the extended hybrid peptide KGNOP1 shows a plausible and comparable binding mode to the active conformation of the MOR. Binding of the NOP receptor-specific substructure to the core region of the MOR is unlikely, due to the more polar character of this peptide part. In contrast, the C-terminal peptide part of KGNOP1 binds to the core region at the NOP receptor. The NOP receptor-specific substructure was found to bind at the core region of the NOP receptor. Since no active NOP receptor conformation has been determined to date, the precise agonist binding mode remains unknown. However, based on our proposed binding mode of KGNOP1 at an inactive NOP receptor conformation, we suggest that the high flexibility of the NOP-specific substructure is likely to be compatible with and bind similarly to an active receptor conformation. Given the size of the ligands, it is highly likely that the non-interacting peptide pharmacophores in KGNOP1 stick out of the binding site and are located in the extracellular receptor region. In consequence, these might be very flexible and able to adopt several conformations. We suggest that the unspecific peptide part contributes less to the pharmacological characteristics at a distinct receptor, and thus provides a way to combine peptide fragments with different functionality. The here-proposed binding modes of KGNOP1 are in line with the pharmacological results and illustrate a plausible active MOR-KGNOP1 and a NOP receptor-KGNOP1 complex (Figure 5).

## 3. Discussion 

Despite awareness of the present opioid epidemic, and the multiple and related severe adverse effects, opioids are still commonly prescribed for the management of acute and chronic severe pain. Hence, the imperative need for safer and non-addictive pain therapies continues to drive the pursuit of novel lead molecules and new treatment strategies [3]. Furthermore, the complexity of chronic pain syndromes requires tailored pharmacological interventions and efficient drugs to fully control pain [10,33].

A promising mechanism-based treatment approach targets multifunctional ligands possessing activity on multiple opioid/non-opioid receptors involved in pain modulation, while reducing undesired side effects [6,8,18,19,20]. Moreover, opioid peptide-derived analgesics are appraised as potential pain therapeutics with lower toxicity [17,34,35,36]. The two strategies are merged in the new opioid peptide-based hybrid, KGNOP1. A major finding of the present study is that KGNOP1 produces potent, effective antinociceptive effects in mouse models of acute and chronic inflammatory pain after s.c. administration, without the typical MOR liabilities of morphine, including rewarding and withdrawal effects. Furthermore, we showed that KGNOP1 possesses full agonist activity for both MOR and NOP receptors in vitro and in vivo, and thus revealed further knowledge into KGNOP1′s mode of action. Additionally, the first in silico structure-based study aided by docking of KGNOP1 to the structures of the MOR and NOP receptors offers important insights into its binding mode, and expands understanding of peptide ligand–OR interactions, which are of significant relevance for the future design of peptide-based analgesics.

Using behavioral studies, our current results established KGNOP1′s efficacy in attenuating pain behavior in mice after s.c. administration in a time- and dose-dependent manner in the formalin test, as a model of tonic inflammatory pain, and in the CFA-induced paw hyperalgesia, as a model of persistent inflammatory pain. Notably, KGNOP1 was more effective in causing an antinociceptive response than morphine. In the formalin test, the antinociceptive effect of KGNOP1 was more marked in the inflammatory Phase II than in the acute nociceptive Phase I at doses of 0.49 and 1.22 µmol/kg (Figure 2A,C,D and Table S1). Determination of the ED_50_ values further revealed that KGNOP1 was 2-fold more potent in the inflammatory phase than in the acute nociceptive phase (Table S1). Furthermore, in the inflammatory Phase II of the formalin test, KGNOP1 was 12-fold more potent than morphine (ED_50_ = 0.55 vs. 6.44 µmol/kg, respectively). This increased potency may be explained by the much higher affinity and agonist potency of KGNOP1 to the MOR in comparison to morphine (K_i_ = 0.42 nM and EC_50_ = 2.50 nM for KGNOP1, Table 1, vs. K_i_ = 3.35 nM and EC_50_ = 34.4 nM for morphine [26]). 

Characteristic differences were observed in the time-course of the antinociceptive effect of KGNOP1 and morphine in mice with CFA-induced chronic inflammatory pain. In contrast to morphine, which produced antinociception of relatively short duration (peak effect at 30 min and rapid decline), KGNOP1 efficiently reversed CFA-induced thermal hyperalgesia with a significantly prolonged effect lasting up to 6 h. The extended effect of KGNOP1 correlates with its reported half-life time (t_1/2_ = 586 min) in human plasma [22]. The high enzymatic stability of KGNOP1 is due to the presence of a *D*-amino acid (*D*-Arg^2^) and unnatural and unusual amino acids (Aba^3^ and β-Ala^4^) [22]. The current in vivo findings on the enhanced and sustained antinociception of KGNOP1 compared to morphine in inflammatory pain following s.c. administration complements previous pharmacological results in acute nociceptive (tail-flick test) and neuropathic pain (diabetic neuropathy and chronic constriction injury of the sciatic nerve) after intrathecal (i.t.) and intravenous (i.v.) injection in mice and rats [22,23,24].

KGNOP1 was initially reported as a bifunctional MOR agonist/NOP receptor antagonist [22]. Whereas our current results endorsed the earlier reported data on the selective and potent MOR agonism of KGNOP1 in vitro [22], we also identified a full agonistic activity to the human NOP receptor, albeit with very low potency in the functional [^35^S]GTPγS binding assay. This result opposes the previous report on the antagonist profile of KGNOP1 to the NOP receptor (pA_2_ = 5.39) in the forskolin-stimulated 3′,5′-cyclic adenosine monophosphate (cAMP) assay using human embryonic kidney (HEK293) cells stably expressing the human NOP receptor [22]. The observed differences in the functional activity of KGNOP1 might be due to the different assays used ([^35^S]GTPγS binding vs. cAMP accumulation), and/or different levels of the NOP receptor expression in CHO vs. HEK293 cells.

Therefore, in vivo antagonism experiments were undertaken in this study to elucidate the contribution of the NOP receptor and MOR agonism to the antinociceptive effect of KGNOP1. We established that the antinociceptive activity of KGNOP1 in the formalin test after s.c. administration was reversed by pre-treatment with the selective NOP receptor antagonist SB-612111 [30] and the MOR preferring antagonist naltrexone [29], indicating that both NOP receptor and MOR agonism are involved in KGNOP1′s in vivo agonist activity. Our findings on the dual MOR/NOP receptor agonism of KNOP1 in mice with inflammatory pain confirmed previous observations in a rat model of neuropathic pain (chronic constriction injury), where pre-treatment with the [*N*phe^1^]-nociceptin (1-13)-NH_2_ (a selective NOP receptor antagonist) and naloxone (an opioid receptor antagonist) blocked the inhibition in pain behavior of KGNOP1 to thermal and mechanical stimulation [24].

In addition to analgesia, activation of the MOR leads to respiratory depression, sedation/locomotor dysfunction, reward/euphoria, and dependence/withdrawal [1]. Herein, behavioral studies with KGNOP1 in mice after s.c. administration demonstrated the absence of detrimental effects of established MOR agonists, particularly their addictive and dependence liabilities. We showed that unlike morphine, chronic s.c. treatment of mice with KGNOP1, at antinociceptive doses effective in inflammatory pain or even at higher doses, caused neither acquisition of CPP nor withdrawal effects and did not affect spontaneous locomotor activity. The lack of KGNOP1′s effect on the evoked locomotor activity in the rotarod test was previously reported following acute i.t. administration in rats [24]. In contrast, we showed herein that morphine produced significant hyperlocomotion in mice in the open-field test, an observation which is in line with published reports [37,38,39,40,41]. The hyperactivity response of morphine is attributed to the activation of the MOR in the mesolimbic dopaminergic system in the nucleus accumbens [37,39]. Recently, Huang et al. also demonstrated using the open-field test that morphine-treated wild-type mice traveled longer distances than humanized MOR mice, which exhibited sedation, similar to observations in the clinical setting [41].

Our current results indicate that KGNOP1 displays a superior benefit/side effect ratio regarding addiction, physical dependence, and locomotor impairment compared to morphine. We demonstrated the absence of these opioid-mediated side effects at a dose shown to be highly effective in inhibiting pain behavior (1.22 µmol/kg, significantly reduced pain behavior in the formalin test and CFA-induced chronic inflammatory pain), but we also showed its safety profile at a higher dose of 2.44 µmol/kg. Demonstrating the safety of a drug in case of an overdose is essential for potential further development

In addition, previous studies reported significantly less respiratory depression of KGNOP1 in contrast to morphine when given i.v. at equianalgesic doses to rats [22,23], pointing to a safer profile of KGNOP1 in terms of this acute side effect. Respiratory safety is of major importance to clinicians due to the risk of fatal outcomes and because respiratory suppression is the primary cause of opioid-related overdose mortality [2]. All these side effects arise from opioid actions in the brain. Respiratory depression results from the activation of MOR in the brainstem medulla, pons, and cortical areas [42]. Reward and dependence mediated by the MOR involve the mesolimbic pathway (ventral tegmental area, nucleus accumbens), amygdala, cortex, and hippocampus [5], while sedation is caused by the MOR activation in the hypothalamic arousal system [43]. Interestingly, although KGNOP1 was reported to cross the blood–brain barrier by active or adsorptive mechanisms [22], in this study, we showed that it has a superior safety profile to morphine concerning addiction, physical dependence, and locomotor impairment, in addition to the earlier observations on reduced respiratory depression [22,23].

Since the discovery of the NOP receptor as the fourth member of the opioid receptor family, its functional role in pain processing with the development of potential pain therapeutics has been increasingly explored [44,45]. The MOR and NOP receptors share common signaling pathways and are both functionally expressed in pain pathways [46,47]. Furthermore, the heterodimerization of the MOR and NOP receptors was reported [48,49], and the design of dual MOR/NOP ligands was undertaken as a promising strategy to bypass the harmful effects of the conventional MOR [20,50]. The most advanced among these compounds, cebranopadol, a small molecule and a bifunctional ligand with full agonist effects at MOR and NOP receptors, is now in advanced clinical development for the treatment of acute and chronic pain [44,51]. Other mixed MOR/NOP receptor ligands showing promising profiles in preclinical studies include other small molecules, such as SR16435 and AT-121 as partial agonists at both receptors. SR16435 induced CPP in mice to the same extent as morphine, suggesting that partial NOP agonism might not be enough to attenuate the rewarding properties associated with the MOR activation [52,53]. In non-human primates, AT-121 produced antinociception with reduced respiratory depression and physical dependence [54]. We propose that, similar to cebranopadol [44,51], the NOP receptor full agonism of KGNOP1 is able to positively suppress the reinforcement acquisition induced by the MOR agonism, as KGNOP1 was devoid of reinforcing effects, withdrawal syndrome, and locomotor dysfunction, shown in this study, as well as a lower propensity for respiratory depression when given systemically to rodents [22,23].

Notably, whereas some mixed MOR-NOP receptor peptide ligands were reported in the literature as potential analgesics [55,56,57,58], their antinociceptive properties were described only in acute thermal nociception (tail-flick test) when injected i.t. or i.c.v. to animals. In contrast, KGNOP1 displays broad activity in various pain states and is highly potent and efficacious in rodent models of acute nociceptive, acute and chronic inflammatory, and chronic neuropathic pain after systemic (i.v. or s.c.) administration.

Substantial advances in the structural biology of GPCRs, including the opioid receptors, together with the availability of efficient computational methods, provide essential insights into ligand binding to the receptor. The gained mechanistic knowledge fosters the structure-based discovery of new opioid receptor modulators [59,60]. Herein, we proposed binding modes of KGNOP1 to the MOR and NOP receptors, which are in line with the pharmacological data. The subtype-specific subsequences, H-Dmt-*D*-Arg-Aba-β-Ala-NH_2_ for the MOR and H-Arg-Tyr-Tyr-Arg-Ile-Lys-NH_2_ for the NOP receptor, bind to the inner core region of the respective receptor, and thereby govern the binding orientation of the whole peptide. The observation of plausible complexes of the MOR and the NOP receptor with KGNOP1 supports the multifunctional characteristics of KGNOP1. Our data also indicated the central contribution of the Dmt moiety for binding to the MOR through a charge interaction with D^3.32^, lipophilic contacts with Y^3.33^ and M^3.36^, and a hydrogen bond to I^6.51^ (Figure 5D), as critical residues for the binding of peptides, morphinans, and other chemotypes to the receptor [32,61,62,63,64]. For binding to the NOP receptor, the D^3.32^ forms charge interactions with the basic Arg^8^ and Lys^10^ of KGNOP1 (Figure 5E).

## 4. Materials and Methods

### 4.1. Drugs and Chemicals

KGNOP1 was prepared as previously described [22]. Cell culture media and supplements were obtained from Sigma-Aldrich Chemicals (St. Louis, MO, USA). Radioligands [^3^H][*D*-Ala^2^,*N*-Me-Phe^4^,Gly-ol^5^]enkephalin ([^3^H]DAMGO, 50 Ci/mmol), [^3^H]diprenorphine (37 Ci/mmol), [^3^H]U69,593 (60 Ci/mmol), [^3^H]Nociceptin (119.4 Ci/mmol), and guanosine 5′-O-(3-[^35^S]thio)-triphosphate ([^35^S]GTPγS, 1250 Ci/mmol) were purchased from PerkinElmer (Boston, MA, USA). Guanosine diphosphate (GDP), GTPγS, DAMGO, [*D*-Pen^2^,*D*-Pen^5^]enkephalin (DPDPE), U69,593, diprenorphine, nociceptin, tris(hydroxymethyl) aminomethane (Tris), 2-[4-(2-hydroxyethyl)piperazin-1-yl]ethanesulfonic acid (HEPES), polyethylenimine (PEI), formalin, and Complete Freund’s Adjuvant (CFA) were obtained from Sigma-Aldrich Chemicals (St. Louis, MO, USA). SB-612111 was obtained from Bio-Techne (Abigdon, UK). Morphine hydrochloride was obtained from Gatt-Koller GmbH (Innsbruck, Austria). PathHunter detection reagents were obtained from DiscoveRx (Birmingham, UK). Naltrexone hydrochloride and naloxone hydrochloride were kindly provided by Helmut Schmidhammer (University of Innsbruck, Innsbruck, Austria). All other chemicals were of analytical grade and obtained from standard commercial sources. Test compounds were prepared as 1 mM stocks in water for in vitro assays or dissolved in physiological 0.9% saline solution for in vivo testing, and further diluted to working concentrations in the appropriate medium.

### 4.2. Cell Cultures and Membrane Preparation

CHO cells stably expressing the human opioid receptors (CHO-hMOR, CHO-hDOR, CHO-hKOR, and CHO-hNOP cell lines) were kindly provided by Lawrence Toll (SRI International, Menlo Park, CA, USA). CHO-hMOR and CHO-hDOR cells were grown at 37 °C in Dulbecco’s Modified Eagle’s Medium (DMEM/Ham’s F12) culture medium supplemented with 10% fetal bovine serum (FBS), 0.1% penicillin/streptomycin, 2 mM L-glutamine, and 0.4 mg/mL geneticin (G418). CHO-hKOR and CHO-hNOP cells were grown at 37 °C in DMEM culture medium supplemented with 10% FBS, 0.1% penicillin/streptomycin, 2 mM L-glutamine, and 0.4 mg/mL geneticin (G418). All cell cultures were maintained in a humidified atmosphere of 95% air and 5% CO_2_. Membranes from CHO-hOR cells were prepared as previously described [26], and stored at −80 °C until use. The protein content of cell membrane preparations was determined by the Bradford method using bovine serum albumin as the standard [65].

### 4.3. Competitive Radioligand Binding Assays for Opioid Receptors

Competitive binding assays were conducted on human opioid receptors stably transfected into CHO cells according to the published procedures [26,27]. Binding assays were performed using [^3^H]DAMGO (1 nM), [^3^H]diprenorphine (0.2 nM), [^3^H]U69,593 (0.4 nM), or [^3^H]Nociceptin (0.1 nM) for labeling MOR, DOR, KOR, or NOP receptors, respectively. Non-specific binding was determined using 1–10 µM of the unlabeled counterpart of each radioligand. Assays were performed in 50 mM Tris-HCl buffer (pH 7.4) in a final volume of 1 mL. In NOP receptor experiments, 1 mg/mL bovine serum albumin (BSA) was added to the assay buffer. Cell membranes (15–20 µg) were incubated with various concentrations of test compounds and the appropriate radioligand for 60 min at 25 °C. After incubation, reactions were terminated by rapid filtration through Whatman GF/C glass fiber filters. In the NOP receptor assay, filtration was carried out through 0.5% PEI-soaked Whatman GF/C glass fiber filters. Filters were washed three times with 5 mL of ice-cold 50 mM Tris-HCl buffer (pH 7.4) using a Brandel M24R cell harvester (Gaithersburg, MD, USA). Radioactivity retained on the filters was counted by liquid scintillation counting using a Beckman Coulter LS6500 (Beckman Coulter Inc., Fullerton, CA, USA). All experiments were performed in duplicate and repeated three times with independently prepared samples.

### 4.4. [^35^S]GTPγS Binding Assays for Opioid Receptors

Binding of [^35^S]GTPγS to membranes from CHO stably expressing the human opioid receptors was conducted according to the published procedure [26]. Cell membranes (5–10 µg) in Buffer A (20 mM HEPES, 10 mM MgCl_2_, and 100 mM NaCl, pH 7.4) were incubated with 0.05 nM [^35^S]GTPγS, 10 µM GDP and various concentrations of test compounds in a final volume of 1 mL for 60 min at 25 °C. Non-specific binding was determined using 10 µM GTPγS, and the basal binding was determined in the absence of test ligand. Samples were filtered over Whatman GF/B glass fiber filters and counted as described for competitive binding assays. All experiments were performed in duplicate and repeated three times with independently prepared samples.

### 4.5. Animals and Drug Administration

Experiments were performed in male CD1 mice (8–10 weeks old, 30–35 g body weight) purchased from Janvier Labs (Le Genest-Saint-Isle, France). All animal care and experimental procedures were in accordance with the ethical guidelines for the animal welfare standards of the European Communities Council Directive (2010/63/EU) and were approved by the Committee of Animal Care of the Austrian Federal Ministry of Science and Research. Mice were group-housed in a temperature-controlled specific pathogen free room with a 12 h light/dark cycle and with free access to food and water. Test compounds were administered by s.c. route in a volume of 10 µL/g body weight. The doses of the investigated compounds were for KGNOP1: 0.12, 0.49, 1.22, and 2.44 µmol/kg (0.25, 1, 2.5, and 5 mg/kg, respectively), and for morphine: 3.11, 7.77, and 15.5 µmol/kg (1, 2.5, and 5 mg/kg, respectively). Upon the completion of the experiments, mice were euthanized by inhalation of carbon dioxide.

### 4.6. Formalin-Induced Acute Inflammatory Pain

The formalin test, originally described by Dubuisson and Dennis [28], was used as a model of tonic inflammatory pain in mice. Following a habituation period of 15 min to individual transparent observation chambers, mice were s.c. administered different doses of the test compound or saline (control), 30 min (KGNOP1) or 15 min (morphine) prior injection of 20 µL of 5% formalin aqueous solution to the plantar surface of the right hindpaw. Each mouse was observed for 60 min in 5 min intervals after the injection of formalin. The amount of time (in seconds, sec) each animal spent licking, biting, lifting, and flinching of the formalin-injected paw (pain behavior) was recorded during Phase I (0–5 min) and Phase II (15–60 min). Antinociceptive effect was calculated according to the following formula: 100 × [(*C* − *T*)/*C*], where *C* is the mean time in control (saline) group and *T* is the time in drug-treated group. In the antagonism studies, naltrexone (1 mg/kg, 2.6 µmol/kg) and SB-612111 (3 mg/kg, 6.6 µmol/kg) were injected s.c. 15 min and 30 min, respectively, before administration of KGNOP1 (1.22 µmol/kg, s.c.) to mice. Doses and pre-treatment times of the antagonists were selected based on previous research [61,66].

### 4.7. Complete Freund’s Adjuvant (CFA)-Induced Chronic Inflammatory Pain

Unilateral CFA-induced inflammation of the hind limb with development of hyperalgesia was used as a model of chronic inflammatory pain in mice [31]. Mice were injected with 20 µL emulsified CFA (1 mg/mL) into the plantar surface of the right hindpaw under brief isoflurane anesthesia. Nociceptive testing was performed 72 h after inoculation with CFA, where mice were accustomed to each testing condition for 2–3 days prior to the inoculation with CFA, using the Hargreaves and von Frey tests. Hindpaws withdrawal latencies to thermal and mechanical stimulation were measured before inoculation with CFA into the mouse right hindpaw (basal latencies, BL), 72 h post-inoculation (pre-treatment values, defined as 0 h), and at 30 min and every hour up to 8 h and 24 h after s.c. administration of saline (control), KGNOP1 (0.49 and 1.22 µmol/kg), or morphine (15.5 µmol/kg).

Thermal sensitivity was assessed using the Hargreaves test [67] with an analgesiometer (Ugo Basile S.R.L., Varese, Italy). Mice were placed in individual Plexiglas boxes positioned on a glass surface (Ugo Basile S.R.L., Varese, Italy). Measurements of paw withdrawal latency to heat stimuli started after a period of habituation of 30 min. A movable infrared generator (30% intensity) located under the glass floor was focused onto the plantar surface of the hindpaw and switched on to heat. Onset of the radiant stimulus triggered a timer which was stopped by subsequent paw movement. Three measurements were carried out and the average value was calculated. To prevent tissue damage, a cut-off time of 20 s was imposed. The latency to withdraw the hindpaw from the stimulus was expressed as paw withdrawal latencies in seconds.

Mechanical sensitivity was assessed by measuring the paw withdrawal threshold in response to probing of the plantar surface of the hindpaw with von Frey filaments on the basis of the up-down method [68]. A series of calibrated von Frey monofilaments with bending forces between 0.04 and 4 g (Ugo Basile S.R.L., Varese, Italy) were used. Mice were placed in individual Plexiglas boxes placed on an elevated platform with a mesh floor. The von Frey filament was inserted perpendicularly from below the platform, up through the wire mesh, and applied to the plantar surface of the hindpaw for 3 s until a behavioral reaction was observed. The behavioral assessment was initiated with a force of 0.6 g and the response as paw withdrawal, shaking, lifting, and licking was recorded. Data were calculated using the Up-Down Reader Software and plotted as the 50% threshold (g) [69].

Antinociceptive effects were determined as percentages (% reversal of thermal/mechanical sensitivity) according to the following formula: 100 × [(T_1_ − T_0_)/(T_BL_ − T_0_)], where T_0_ is the nociceptive value at 72 h post-inoculation with CFA (defined as 0 h), T_1_ is the value obtained following drug administration, and T_BL_ is the basal value before CFA inoculation.

### 4.8. Open-Field Test

The open-field test was used for evaluating the drug effect on spontaneous locomotor activity [70]. The global locomotor activity was measured using custom-made chambers (height × width × height, 30 × 30 × 25 cm) in an isolated noise-free room illuminated to 60 Lux. On the experimental day, mice were s.c. administered saline (control), KGNOP1, or morphine and placed in the chambers, and locomotor activity was video-recorded for 30 min using the EthoVision XT system (Noldus Information Technology, Wageningen, The Netherlands). Locomotion was assessed daily for four consecutive days after chronic test drug or saline (control) administration, during the conditioning sessions in the CPP test described below. The traveled distance (in cm) during each test day was determined.

### 4.9. Conditioned Place Preference

Conditioned place preference (CPP) was used to evaluate rewarding drug effects in mice [71]. The CPP test was conducted in a custom-made, three-chamber apparatus (two conditioning compartments separated by a neutral chamber) [27] with some modifications as described below. All experiments were performed in an isolated noise-free room illuminated to 60 Lux. The protocol was performed in three phases. (1) In the preconditioning phase, drug-naive mice were placed in the center compartment and the doors were opened, so both test chambers were accessible, and the time spent in each chamber was recorded for 15 min. (2) In the conditioning phase, which lasted 4 days, the conditioning chambers were closed. In the morning of the each conditioning day, mice received saline and were placed individually for 30 min in the test chamber that the particular mouse spent the most amount of time in during the pretest phase. In the afternoon (4 h later), mice were given the drug and confined for 30 min in the opposite compartment. This sequence was alternated over the next 3 days. The control mice received saline in both compartments during conditioning. Drug-treated mice received saline in the morning and KGNOP1 (1.22 or 2.44 µmol/kg) or morphine (15.5 µmol/kg) in the afternoon. (3) In the test phase, mice were placed in the center chamber and allowed to freely move in the entire CPP apparatus. No drug or vehicle was given on this day and the location of the mouse was measured for 15 min using EthoVision XT system (Noldus Information Technology, Wageningen, The Netherlands). CPP results were calculated as the difference in the time spent in the drug-paired compartment during the test phase and the time spent in the same compartment during the preconditioning phase.

### 4.10. Naloxone-Precipitated Withdrawal Syndrome

Opioid physical dependence was assessed using a precipitated withdrawal approach in mice as described previously [21] with some modifications. Mice received daily s.c. injections of KGNOP1, morphine, or saline over a 5-day period. On day 5, two hours after the last drug injection, the withdrawal syndrome was precipitated by administration of naloxone (1 mg/kg, s.c.). Animals were immediately placed in clear acrylic cylinders, and signs of opioid withdrawal were recorded for 15 min. Monitored behaviors included vertical jumps, paw tremor, head shakes, urine output, presence/absence of diarrhea, number of droppings, and body weight loss. For each mouse, a global withdrawal score was calculated by summing the values obtained for each sign (one point was assigned to every 3 jumps and 5 paw tremors, whereas all other signs were given the absolute values recorded during the test).

### 4.11. Molecular Modeling

Potential binding modes of KGNOP1 to the MOR and NOP receptors were evaluated by molecular docking using CCDCs software GOLD version 5.7.0 [72]. A humanized MOR model based on the active cryo-electron microscopy (cryo-EM) structure of the mouse MOR (PDB entry: 6DDF, chain R) [12] was used for docking of KGNOP1 to the MOR taking KGOP01 as reference ligand as previously reported [32]. The inactive crystal structure of the human NOP receptor (PDB entry: 5DHG, chain A) [25] was used for docking KGNOP1 to the NOP receptor, since no active receptor conformation has been reported yet. We used the binding site of the co-crystallized NOP antagonist (PDB entry: DGV; (1-benzyl-*N*-{3-[4-(2,6-dichlorophenyl)piperidin-1-yl]propyl}-*D*-prolinamide)) for docking. All residues of the inner receptor core region and the extracellular loop region were defined as potential binding site for both receptors. Water molecules and ligands were removed prior to docking and protonation states were assigned using Protonate3D [73] (implemented in MOE 2019.0102, Chemical Computing Group, Montreal, QC, Canada). Atom coordinates for KGNOP1 were obtained with the molecule builder and subsequent energy minimization (MMFF94x) in MOE (MOE 2019.0102, Chemical Computing Group, Montreal, QC, Canada). Docking poses were analyzed with LigandScout 4.2 using a 3D-pharmacophore approach [74,75], with particular emphasis on a charge interaction with the key D^3.32^ residue.

### 4.12. Data and Statistical Analysis

Experimental data were graphically processed and statistically analyzed using the GraphPad Prism 5.0 Software (GraphPad Prism Software Inc., San Diego, CA, USA). In in vitro assays, inhibition constant K_i_ (nM), potency EC_50_ (nM), and efficacy E_max_ (%) values were determined from concentration-response curves by nonlinear regression analysis. In competitive radioligand binding assays, data were normalized to the percentage of specific binding of the respective radioligand. The K_i_ values were determined by the method of Cheng and Prusoff [76]. In the [^35^S]GTPγS binding assays, efficacy was determined relative to the reference full agonists, DAMGO (MOR), DPDPE (DOR), U69,593 (KOR), or nociceptin (NOP receptor). All in vitro experiments were performed in multiple replicates with at least three independent experiments unless otherwise indicated. In the formalin test, dose-response relationships of pain scores were constructed, and the dose necessary to produce a 50% effect (ED_50_) and 95% confidence limits (95% CL) were calculated according to the method of Litchfield and Wilcoxon [77]. In the CCP assay, data are presented as time spent in the drug-paired compartment post-test minus pre-test. For in vivo behavioral data, two-sample comparison was performed using a *t*-test. For multiple comparisons between the treatment groups, ANOVA (one-way or two-way, as appropriate) with Dunnett’s or Bonferroni’s post hoc tests were used. All data are presented as mean ± SEM. A *p* < 0.05 was considered statistically significant.

## 5. Conclusions

In conclusion, the current pharmacological data indicate that the dual MOR/NOP receptor hybrid peptide KGNOP1 produces potent and long lasting antinociception in mouse models of inflammatory pain after systemic s.c. administration. Despite its limited NOP receptor over MOR selectivity in vitro, receptor antagonist in vivo studies established that the antinociceptive effects of KGNOP1 are mediated via the MOR and NOP receptor activation. Furthermore, KGNOP1, by its combination of agonism at the MOR and NOP receptors, affords higher antinociception potency and prolonged efficacy compared to morphine, and shows reduced MOR-mediated liabilities, particularly addiction, physical dependence, and locomotor impairment following acute and repeated/chronic s.c. administration, thus a better tolerability profile. In addition, KGNOP1 is the only mixed MOR/NOP receptor peptide-based agonist known to date, which has been demonstrated to be effective in a multitude of pain conditions ranging from nociceptive to inflammatory and neuropathic pain in rodents with reduced side effects of conventional opioids. Its favorable pharmacology makes KGNOP1 a potential drug candidate that merits further exploration. A combination of experimental and molecular modeling strategies presents a translational bridge to facilitate research and the development of dual MOR/NOP receptor agonists as improved treatments for various pain conditions.

## Figures and Tables

**Table 1:** Binding affinities and functional activities of KGNOP1 to the human MOR, DOR, KOR, and NOP receptors.

Target	Receptor Binding ^a^	[^35^S]GTPγS Binding ^b^	
	K_i_ (nM)	EC_50_ (nM)	E_max_ (%)
MOR	0.42 ± 0.08	2.50 ± 0.81	92 ± 4
DOR	12.2 ± 3.5	51.6 ± 17.8	106 ± 3
KOR	13.4 ± 4.3	112 ± 32	37 ± 3
NOP	141 ± 23	1391 ± 278	99 ± 8

^a^ Determined in competitive radioligand binding assays using membranes of CHO cell stably expressing the human MOR, DOR, KOR, or NOP receptors (CHO-hOR). Inhibitory constant (K_i_) values were calculated from the competition binding curves by nonlinear regression analysis. ^b^ Determined in the [^35^S]GTPγS binding assays with membranes of CHO cells stably expressing the human opioid receptors. Efficacy (E_max_, %) is expressed as percentage relative to the maximum effect of DAMGO (MOR), DPDPE (DOR), U69,593 (KOR), or nociceptin (NOP receptor) (as 100%). Values are expressed as the mean ± SEM (n = 3–4 independent experiments performed in duplicate).

## Data Availability

Data are contained within the article.

## Supplementary Materials

The following are available online, Table S1: Antinociceptive effect of KGNOP1 and morphine after s.c. administration in the formalin test in mice; Table S2: Antinociceptive effect of KGNOP1 and morphine after s.c. administration to mice with CFA-induced inflammatory pain; Figure S1: Effect of KGNOP1 and morphine on naloxone-precipitated withdrawal syndrome in mice after s.c. administration.

## Sample Availability

Sample of the compound is available from the authors.
