# Supplementary material for: Antinociceptive Efficacy of the µ-Opioid/Nociceptin Peptide-Based Hybrid KGNOP1 in Inflammatory Pain without Rewarding Effects in Mice: An Experimental Assessment and Molecular Docking
Source: Molecules. 2021 May 28;26(11):3267. doi: 10.3390/molecules26113267 (PMC8198056; doi:10.3390/molecules26113267)
Supplement: Supplementary file 1 [file molecules-26-03267-s001.zip › molecules-1210249-supplementary.pdf]

Antinociceptive efficacy of the µ-opioid/nociceptin peptide-based hybrid KGNOP1 in inflammatory pain without rewarding effects in mice: An experimental assessment and molecular docking

Maria Dumitrascuta[1], Marcel Bermudez[2], Olga Trovato[1], Jolien De Neve[3], Steven Ballet[3], Gerhard Wolber[2] and Mariana Spetea[1,*]

[1] Department of Pharmaceutical Chemistry, Institute of Pharmacy and Center for Molecular Biosciences Innsbruck (CMBI), University of Innsbruck, Innrain 80-82, 6020 Innsbruck, Austria
[2] Institute of Pharmacy, Freie Universität Berlin, Königin-Luise-Str. 2+4, D-14195 Berlin, Germany
[3] Research Group of Organic Chemistry, Departments of Chemistry and Bioengineering Sciences, Vrije Universiteit Brussel, Pleinlaan 2, B-1050 Brussels, Belgium

***Correspondence:**
Mariana Spetea
Department of Pharmaceutical Chemistry, Institute of Pharmacy and Center for Molecular Biosciences (CMBI), University of Innsbruck, Innrain 80-82, 6020 Innsbruck, Austria; Tel.: +43 512 507 58277; Fax: +43 512 507 58299; e-mail: mariana.spetea@uibk.ac.at

Table S1. Antinociceptive effect of KGNOP1 and morphine after s.c. administration in the formalin test in mice

**Treatment**<br>**Dose**	**Phase I**<br>**%Antinociceptive effect**	**Phase II**<br>**%Antinociceptive effect**
KGNOP1		
0.12 μmol/kg	33.9 ± 3.8	2.3 ± 11.1
0.49 μmol/kg	33.7 ± 9.3	51.8 ± 9.7
1.22 μmol/kg	55.6 ± 7.3	71.4 ± 6.8
ED_{50} (μmol/kg) (95% CL)	1.17 (0.41-3.35)	0.55 (0.22-1.33)
Morphine		
3.11 μmol/kg	19.4 ± 6.7	24.8 ± 11.6
7.77 μmol/kg	32.6 ± 5.7	57.5 ± 8.3
15.5 μmol/kg	38.4 ± 10.9	79.1 ± 7.5
ED_{50} (μmol/kg) (95% CL)	-[a]	6.44 (3.20-12.7)

Data are shown as percent (%) antinociceptive effect calculated for the acute nociceptive pain (Phase I, 0-5 min) and inflammatory pain (Phase II, 15-60 min) as: $100 \times [(C - T)/C]$, where C is the mean time in control (saline) group and T is the time in drug-treated group. The antinociceptive effective dose (ED_{50}) and 95% confidence limit (CL) values were calculated using linear regression. -[a] not calculable. Values represent the mean ± SEM (n = 6-8 mice per group).

Table S2. Antinociceptive effect of KGNOP1 and morphine after s.c. administration to mice with CFA-induced inflammatory pain

Treatment Dose	30 min	1 h	2 h	3 h	4 h	5 h	6 h	7 h	8 h	24 h
**Thermal sensitivity (Hargreaves test)**										
KGNOP1 0.49 µmol/kg	23.5 ±12.0	38.1 ±12.5	59.8 ±16.2	40.2 ±7.7	25.4 ±7.3	15.7 ±8.0	9.3 ±6.0	-1.46 ±4.4	6.12 ±3.5	-3.79 ±4.5
1.22 µmol/kg	31.9 ±11.0	54.4 ±12.3	98.6 ±16.2	78.6 ±11.5	58.2 ±11.2	26.0 ±7.2	26.7 ±9.4	10.2 ±5.2	9.15 ±5.4	6.72 ±3.7
Morphine 15.5 µmol/kg	68.6 ±2.8	36.1 ±3.6	19.6 ±2.8	13.8 ±0.5	-1.96 ±2.3	0.43 ±1.5	-0.40 ±2.6	-	-	0.00 ±0.6
**Mechanical sensitivity (von Frey test)**										
KGNOP1 1.22 µmol/kg	-6.71 ±23.2	65.4 ±21.2	19.1 ±24.1	-8.73 ±31.8	21.1 ±40.6	-0.50 ±25.8	-12.9 ±26.6	-8.30 ±7.3	-2.42 ±26.5	-8.66 ±18.4
Morphine 15.5 µmol/kg	63.4 ±10.2	50.6 ±16.7	18.9 ±7.5	6.62 ±9.6	1.57 ±3.3	11.7 ±6.5	-2.29 ±6.0	-[a]	-[a]	1.68 ±9.0

Data are shown as percentages (%) reversal of thermal/mechanical sensitivity calculated as: $100 \times [(T_1 - T_0)/(T_{BL} - T_0)]$, where T_0 is the nociceptive value at 72 hours post-inoculation with CFA (defined as 0 hours), T_1 is the value obtained following drug administration and T_{BL} is the basal value before CFA inoculation. -[a] not determined. Values represent the mean ± SEM (n = 6-8 mice per group).

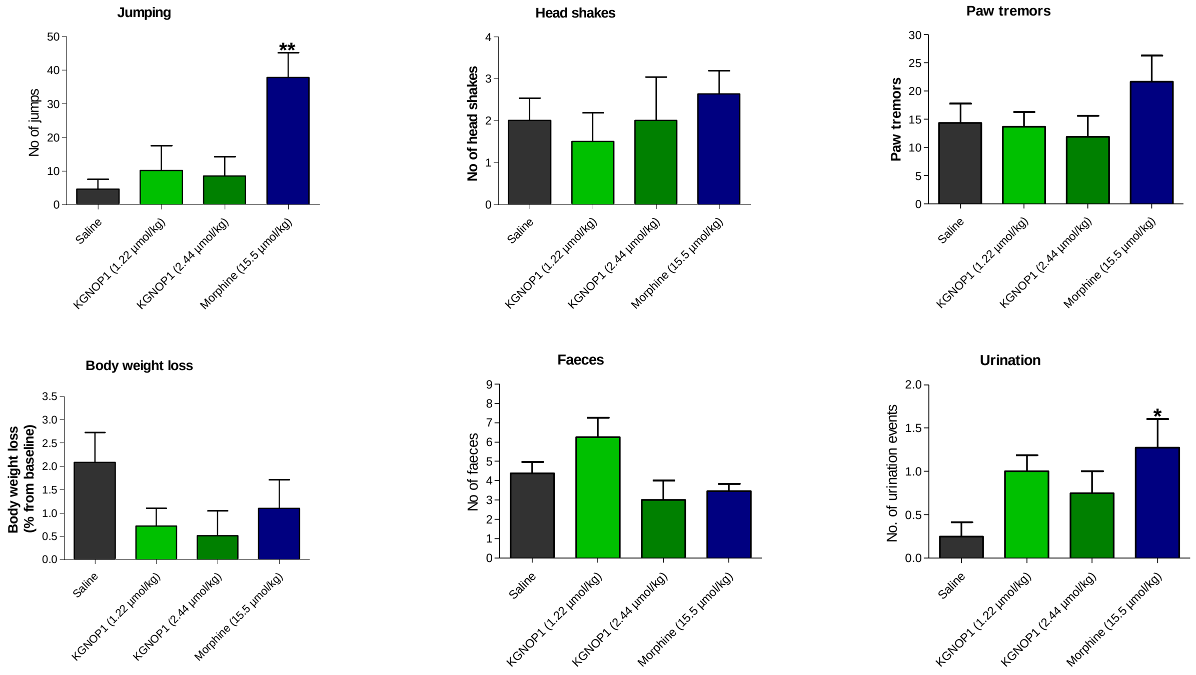

Figure S1. Effect of KGNOP1 and morphine on naloxone-precipitated withdrawal syndrome in mice after s.c. administration. Groups of mice received saline (control), KGNOP1or morphine, daily for 5 days. Naloxone (1 mg/kg, s.c.) was injected two hours after last drug treatment to precipitate withdrawal signs. Number of occurring events were recorded for 15 min after naloxone administration. Data are presented as the mean ± SEM (n = 8 mice per group). *$P < 0.05$ and **$P < 0.01$ vs. saline group, one-way ANOVA followed by Dunnett’s *post-hoc* test.
